# Combined full-length transcriptomic and metabolomic analysis reveals the molecular mechanisms underlying nutrients and taste components development in *Primulina juliae*

**DOI:** 10.1186/s12863-024-01231-z

**Published:** 2024-05-23

**Authors:** Yi Zhang, Endian Yang, Qin Liu, Jie Zhang, Chen Feng

**Affiliations:** 1 Jiangxi Provincial Key Laboratory of ex situ Plant Conservation and Utilization, Lushan Botanical Garden, Chinese Academy of Sciences, Zhiqing Rd, No. 9, Jiujiang, 332900 China; 2 College of Life Science, Nanchang University, Nanchang, China

**Keywords:** Full-length transcriptomic, *Primulina juliae*, Nutrients components, Taste components, Molecular mechanisms

## Abstract

**Background:**

*Primulina juliae* has recently emerged as a novel functional vegetable, boasting a significant biomass and high calcium content. Various breeding strategies have been employed to the domestication of *P. juliae*. However, the absence of genome and transcriptome information has hindered the research of mechanisms governing the taste and nutrients in this plant. In this study, we conducted a comprehensive analysis, combining the full-length transcriptomics and metabolomics, to unveil the molecular mechanisms responsible for the development of nutrients and taste components in *P. juliae*.

**Results:**

We obtain a high-quality reference transcriptome of *P. juliae* by combing the PacBio Iso-seq and Illumina sequencing technologies*.* A total of 58,536 cluster consensus sequences were obtained, including 28,168 complete protein coding transcripts and 8,021 Long Non-coding RNAs. Significant differences were observed in the composition and content of compounds related to nutrients and taste, particularly flavonoids, during the leaf development. Our results showed a decrease in the content of most flavonoids as leaves develop. Malate and succinate accumulated with leaf development, while some sugar metabolites were decreased. Furthermore, we identified the different accumulation of amino acids and fatty acids, which are associated with taste traits. Moreover, our transcriptomic analysis provided a molecular basis for understanding the metabolic variations during leaf development. We identified 4,689 differentially expressed genes in the two developmental stages, and through a comprehensive transcriptome and metabolome analysis, we discovered the key structure genes and transcription factors involved in the pathways.

**Conclusions:**

This study provides a high-quality reference transcriptome and reveals molecular mechanisms associated with the development of nutrients and taste components in *P. juliae*. These findings will enhance our understanding of the breeding and utilization of *P. juliae* as a vegetable.

**Supplementary Information:**

The online version contains supplementary material available at 10.1186/s12863-024-01231-z.

## Background

Calcium (Ca) is crucial for various human metabolic and physiological processes, representing about 2% of total body weight [1]. It plays a vital role in the growth of bones and teeth, helps achieve peak bone mass in the young, and maintains it in older individuals [2]. Moreover, adequate calcium intake is associated with protection against various types of cancer [3, 4], as well as decreased risks of insulin resistance and cardiovascular diseases [5], highlighting its potential health benefits. Although the Food and Agriculture Organization of the United Nations (FAO) recommends daily Ca intake of 800 to 1,300 mg for adults and 1,300 mg for children above 9 years of age, many individuals, especially from low-income regions of Asia, Africa and Latin America, fail to meet the requirement, with average intakes below 400 mg/day [2, 6–8]. Calcium deficiency is common in these regions due to limited food sources and dietary constraints, like food allergies and dyspepsia [9]. This has led to an increased focus on exploring food from plant-based calcium sources to address these nutritional gaps.

*Primulina*, a genus belonging to the Gesneriaceae family, is the largest genus in this family. It is primarily found in the karst areas of southern China and neighboring Southeast Asian countries [10]. Many *Primulina* plants originating from karst limestone areas exhibit high calcium content, and some species are known for their rich bioactive calcium content in their leaves [11–13]. Additionally, due to the rich content of active metabolites like flavonoids, phenylpropanoid glycosides, quinones, terpenoids, some *Primulina* plants are used as folk herbal medicines in traditional Chinese medicine [13–15]. Through a series of screening and cross breeding efforts, our laboratory has strategically domesticated *Primulina* plants in recent years, developing them into calcium-rich vegetables [13, 16, 17]. *Primulina juliae*, characterized by its rapid growth and substantial biomass, serves as one of our primary breeding materials.

In recent years, there has been a growing pursuit of breeding nutrient-rich and flavorful vegetables. Unveiling the key mechanisms underlying the nutrients and taste components will promote the modern crop breeding. The metabolite and associated gene regulation networks of flavor and nutrients have been reported in many fruits and vegetables, by combining the analysis of transcriptomics and metabolomics, such as that in pumpkin (*Cucurbita moschata*) [18], kiwifruit (*Actinidia chinensis*) [19] and vegetable soybean (*Glycine max*) [20]. However, the molecular mechanisms associated with the development of nutrients and taste components in *P. juliae* have not yet been elucidated, which affects its genetic improvement.

In this study, we investigated metabolomes and transcriptomes in the leaves of *P. juliae* at two growth stages to identify differentially accumulated metabolites and differentially expressed genes. Our data reveal the molecular regulatory mechanisms governing the biosynthesis of nutrients and taste components and identify key biosynthesis-related genes. The findings of this study offer a new perspective on understanding the molecular mechanisms associated with the development of nutrients and taste components, as well as potential targets for molecular breeding in *P. juliae*.

## Methods

### Plant materials

The “Mega-leaf 1” cultivar of *P. juliae* was utilized in this study. It boasts a leaf area of up to 84.55 cm^2^, significantly larger than other genotypes by a factor of 1.46 to 2.12. Additionally, it demonstrates enhanced resistance to gray mold and whiteflies in our greenhouse, highlighting its considerable potential for breeding into biomass and pest and disease resistance. The plant samples were cultivated in a research greenhouse located at the Research Center of Lushan Botanical Garden, Chinese Academy of Science, in Jiangxi Province (115.8382°E; 28.9112°N). They were managed with standard and regular agricultural practice. When the plants reached 120 days of growth, exhibiting six pairs of opposite leaves, 12 plants with uniform growth were randomly divided into 4 biological replicate groups for the experiment. Bud leaves (referred to as “Bud”) and mature leaves (referred to as “Leaf”) in each biological replicate group were harvested (Fig. S1). The collected samples were promptly frozen in liquid nitrogen and stored at -80 ℃ until the transcriptomic and metabolic analysis.

### Sample preparation and metabolomics analysis

The extraction, identification and quantification of metabolites were carried out by Wuhan MetWare Biotechnology Co., Ltd. (Wuhan, China). Metabolite extraction and profiling were conducted following the methods previously described by Chen et al. [21]. In brief, freeze-dried leaf samples were processed in a mixer mill (MM 400; Retsch, Haan, Germany) for 90 s at 45 Hz. Subsequently, 50 mg aliquot of each sample was transferred to a centrifuge tube, and 1.2 ml of a 70% methanol solution was added. The mixture was vortexed for 30 s every 30 min. The extraction solution was then centrifuged at 12,000 rpm for 10 min at 4 ℃ to separate the supernatant. The supernatant was further filtered through a 0.22 μm microporous membrane (SCAA-104, 0.22 μm pore size; ANPEL, Shanghai, China). To assess the stability of the measurement process, a quality control (QC) sample was prepared by pooling a portion of each sample. Extracted supernatants were stored at -80 ℃ until they were analyzed using ultra-performance liquid chromatography-tandem mass spectrometry (UPLC-MS/MS).

A UPLC–ESI–MS/MS system (UPLC, SHIMADZU Nexera X2, Shimadzu, Kyoto, Japan; MS, Applied Biosystems 4500 Q TRAP, Waltham, MA, USA) equipped with an Agilent SB-C18 column (1.8 µm, 2.1 mm × 100 mm) was employed to analyze the extraction samples, following a previously established method by Peng et al. [22]. Triple quadrupole scans were acquired by MRM assays with collision gas (nitrogen) set to medium. The declustering potential (DP) and collision energy (CE) for individual MRM transitions were further optimized. Specific sets of MRM transitions were monitored for each period according to the metabolites eluted during that period. Mass spectra were collected and processed using Analyst v1.6.3 (AB Sciex, Foster City, CA, USA).

The generated mass spectra were matched against MWDB database (MetWare, Wuhan, China) and public metabolite databases. Metabolites were identified by comparing the accurate precursor ions, product ions, retention times, and fragmentation patterns of primary and secondary mass spectra. In this study, metabolite annotations were categorized into three levels, as established in a previous study [21]. The chromatographic peak areas were integrated to represent the relative abundance of each metabolite in different samples. Differentially accumulated metabolites (DAMs) were determined based on *P* value < 0.05. Kyoto Encyclopedia of Genes and Genomes (KEGG) enrichment analysis was conducted with the KEGG Orthology software (http://kobas.cbi.pku.edu.cn/ (accessed on 14 June 2023)). Moreover, the key active ingredients were identified based on a search in the Traditional Chinese Medicine Systems Pharmacology Database and Analysis Platform (TCMSP, http://www.tcmsp-e.com (accessed on 25 July 2023)). We used the parameters oral bioavailability (OB) ≥ 30% and drug-likeness (DL) ≥ 0.18 as the screening criteria.

### RNA extraction and transcriptomic analysis

To obtain a high-quality reference transcriptome, single-molecule real-time sequencing was used in this study. Various tissues, including roots, stems, buds, leaves, and flowers from a single *P. juliae* individual, were collected. Total RNA extraction of the samples and complementary DNA (cDNA) library construction were conducted by BioMarker Co., Ltd. (Beijing, China). For each tissue, the short-paired reads were sequenced using the Illumina platform to assist the correction of the long reads. The total RNA from each tissue was mixed in equal amounts to construct libraries for full-length transcriptome sequencing. The library preparations were subsequently sequenced using the Illumina HiSeq6000 and PacBio Sequel platform. The raw data can be accessed from the NCBI Sequence Read Archive (SRA) platform under the accession number PRJNA1032441.

The raw next-generation sequencing (NGS) data were first processed through in-house Perl scripts (Figshare 10.6084/m9.figshare.10185056), which involved the removal of all adapters, poly-N and low-quality reads to obtain clean reads. Raw SMRT sequencing data were processed following the IsoSeq protocol through the SMRT analysis package version 2.3.0 (Pacific Biosciences, https://www.pacb.com/products-and-services/analytical-software/smrt-analysis). Circular consensus sequencing (CCS) reads were generated from subread Binary Alignment Map (BAM) files. The CCS reads were classified into full-length non-chimeric (FLNC) reads and non-full-length reads, based on the observation of the 5’ primer, 3’ primer, and poly-A tail. The full-length sequences were further clustered using ICE to obtain the cluster consensus sequence. NGS data obtained from the same individual were used for mismatch correction with the software LoRDEC version 0.7 [23].

Candidate coding sequences (CDSs) were predicted with TransDecoder v. 5.5.0 [24] in the full-length transcriptome of *P. juliae*. CD-HIT v. 4.7.0 [25] were used to further reduce redundancy of the final predicted CDSs with the sequence identity threshold of 0.80. Gene functional annotation was performed using various databases, including NCBI non-redundant protein sequences (Nr), Protein family (Pfam), SwissProt, KEGG, Gene Ontology (GO) and EggNOG database. Benchmarking universal single-copy orthologs (BUSCO) version 3 [26] was used to assess the quality of final CDSs transcripts.

Long Non-coding RNAs (lncRNAs) were identified using the following pipeline: (1) Transcripts less than 200 nt were removed. (2) The retained transcripts were evaluated for potential protein-coding using CPC2 [27], CNCI [28] and LGC [29], and intersection sequences of the three methods were extracted. (3) The resulting sequences were filtered based on the coding potential and protein domain analysis including the use of Pfam and TransDecoder platforms.

Simple sequence repeat (SSRs) of the transcriptome were identified using MIcroSAtellite (MISA, an identification tool, http://pgrc.ipk-gatersleben.de/misa/), with the following parameters: definition (unit_size, min_ repeats): 1–10 2–6 3–5 4–5 5–5 6–5, and a maximum difference of 100 between two SSRs.

Gene expression levels were calculated by mapping Bud and Leaf samples to the reference transcriptome by Salmon version 1.3.0 [30]. Genes with Fragments Per Kilobase of exon model per Million mapped fragments (FPKM) values less than 1 were filtered out, and the differential analysis of gene expression were conducted using DESeq2, with parameters set to fold change greater than 2 and a false discovery rate (FDR) less than 0.05 [31]. The GO and KEGG enrichment analysis of differentially expressed genes (DEGs) were performed using the clusterProfiler package in R [32].

### Metabolite biosynthesis pathway analysis

The information of metabolite biosynthesis pathways is collected from KEGG (https://www.kegg.jp/ (accessed on 1 July 2023)). Genes associated with these pathways were also identified. The protein sequences for the relevant gene family in *Arabidopsis* were downloaded from TAIR (https:// www.arabidopsis.org/ (accessed on 2 July 2023)). These sequences were used as queries to perform BLAST searches against the protein sequences in *P. juliae* transcriptome database. To confirm the domain information, the candidate genes were characterized using CDD (https://www.ncbi.nlm.nih.gov/Structure/bwrpsb/ bwrpsb.cgi (accessed on 2 July 2023)) and the Pfam database (http://pfam.xfam.org/search (accessed on 2 July 2023)). The sequences lacking conserved domain were manually eliminated. The FPKM values and sequences of the gene family members were extracted for subsequent analysis.

### Quantitative Real-Time PCR (qRT-PCR) validation for the transcriptome data

To assess the reliability of the RNA-seq results, twelve genes with relative expression were selected to examine. The qRT-PCR primer pairs of genes were designed using Primer Premier v5.0 (Premier Biosoft, Palo Alto, CA, USA) (Table S1). qRT-PCR experiments were conducted using the Bio-Rad CFX96 real-time PCR detection system (Hercules, CA, USA) following the conditions outlined below: denaturation at 95 °C for 5 min and 42 cycles of 95 °C for 10 s, 56 °C for 20 s, and 72 °C for 30 s. Three technical and biological replicates were used. The expression level of each gene was calculated relative to the reference gene, *PjuGAPDH*.

### Statistical analysis

Data comparison among samples were performed by one-way analysis of variance (ANOVA), with statistical significance set at *P* value < 0.05. Principal component analysis (PCA), partial least squares discriminant analysis (PLS-DA), heatmap analysis, volcano plot, and enrichment analysis were performed by the R software with FactoMineR, mixOmics, pheatmap, ggplot2, and clusterProfiler packages. Co-expression network was constructed based on Pearson correlation coefficients (PCC). PCC were calculated by the Scipy package in Python. Genes or metabolites that exhibited PCC values greater than 0.95 or less than -0.95 and *P* value less than 0.05 were used to create the co-expression network. The resulting network was visualized using Cytoscape software version 3.7.2 [33]. The random forest analysis was carried out using the randomForest function in R. The top 20 metabolites with the largest differential contributions were selected based on a combination of accuracy and mean decrease in Gini impact.

## Results

### *Statistical* analysis of transcriptome sequencing data

The PacBio Sequel platform was utilized to sequence the mixed samples and to construct a high quality full-length transcriptomic database, as there was no available *P. juliae* reference genome. In summary, the PacBio Sequel platform generated 37,739,726 subreads (Table 1). After integrating subreads and conducting error correction through multiple sequencing, a total of 650,076 CCS reads were obtained, with an average length of 1,765 bp (Table 1 and Fig. S2). These CCS reads were further utilized to identify the FLNC reads. A total of 509,175 reads were identified as FLNC, with an average N50 length of 2,249 bp (Table 1 and Fig. S2). After clustering the FLNC sequences with ICE algorithm, we totally obtained 58,536 cluster consensus sequences, with an average length of 1,409 bp (Table 1 and Fig. S2). Then, we used LoRDEC software to correct SMRT PacBio data with NGS data.

**Table 1:** The summary of reads from PacBio single-molecule long-read sequencing

	Subreads	CCS	FLNC	Cluster
Subread number	37,739,726	650,076	509,175	58,536
Bases	50,626,053,332	1,148,260,301	701,368,199	82,504,679
Average length	1,341	1,765	1,377	1,409
N50	1,744	2,249	1,898	1,930
GC content (%)	40.98	41.65	42.61	42.58

The process of removing redundant protein sequences yielded 28,168 complete protein coding transcripts. To assess the completeness of the transcriptomes, BUSCO was employed to compare complete protein coding transcripts against a set of conserved plant genes in embryophyta_odb10 dataset. The results revealed a total of 1,614 BUSCO groups, with 1,207 (74.8%) categorized as complete BUSCOs, comprising 1,148 single-copy and 59 duplicated BUSCOs. The missing and fragmented BUSCOs were 210 (13.0%) and 197 (12.2%), respectively (Table S2).

**Fig. 1:**
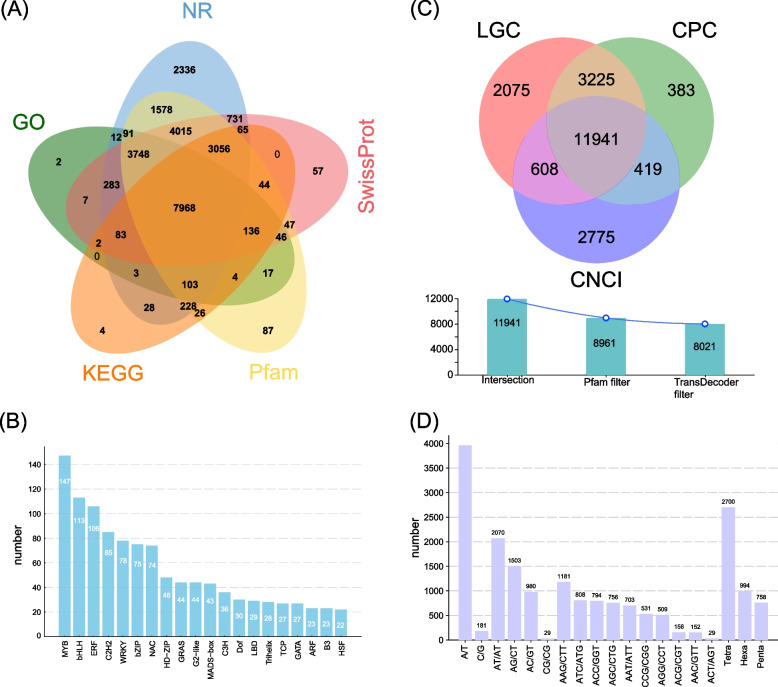
Gene function annotation of coding sequences (CDSs), prediction of transcription factors (TFs), long non-coding RNAs (lncRNAs) and simple sequence repeat (SSRs) in *P. juliae*. **A**Venn diagram of gene function annotation; (**B**) Summary of the numbers of TOP25 TFs classification; (**C**) Number of lncRNAs identified with the CPC, CNCI, LGC, Pfam and TtransDecoder methods; (**D**) Type distribution of SSRs identified

The high-quality full-length transcripts of *P. juliae* were annotated based on the databases of NR, SwissProt, KEGG, Pfam, and GO. A total of 24,807 transcripts (88.1%) were annotated for function, among which 24,328 and 20,228 transcripts were assigned to NR and SwissProt databases, respectively (Fig. 1A). In total, there were 1,366 transcription factors (TFs) were predicted, including 26 families of TFs with a number ranging from 1 to 147. The top 20 abundant TFs included MYB (147), bHLH (113), ERF (106), C2H2 (85), WRKY (78), bZIP (75), NAC (74), HD-ZIP (48), GRAS (44), G2-like (44), MADS-box (43), C3H (36), Dof (30), LBD (29), Trihelix (28), TCP (27), GATA (27), ARF (23), B3 (23), and HSF (22) (Fig. 1B). These TFs collectively accounted for 80.7% of the total TFs (Table S3).

LncRNAs play a crucial role in plant growth, development, metabolism, and stress responses [34–36]. Based on the intersection of the LGC, CPC and CNCI methods, we obtained 11,941 lncRNAs with known protein domains. Finally, 8,021 lncRNAs were predicted by Pfam and Transdecoder filtration (Fig. 1C).

A total of 58,536 transcript were scanned using the MISA software and 13,233 SSR sequences were detected (Table S4). The most prevalent repeat motif type was trinucleotide (5,619), followed by dinucleotide (4,582) and mononucleotide (4,142) (Table S4). Among single repeat motifs, SSR composed of A/T were more abundant than those containing G/C. The AT type SSRs were the most abundant in dinucleotide repeat motif, constituting 45.2% (2,070) of the total in dinucleotide repeat motifs. Ten different types of trinucleotide SSRs were detected, with the number varying from 29 to 1,181 (Fig. 1D). A total of 2,700, 758 and 994 SSRs were identified with tetranucleotide, pentanucleotide and hexanucleotide, respectively (Table S4 and Fig. 1D).

### Transcriptomic changes from bud to leaf

To identify the key genes associated with metabolite biosynthesis pathway, NGS was performed on both Bud and Leaf samples. After filtration, a total of 582,648,772 clean reads were generated. The Q30 percentage and GC percentages ranged from 94.91% to 95.98% and 44.93% to 46.52%, respectively, across the eight libraries. These clean reads were subsequently mapped to the reference transcriptome, with 58.22 to 60.73 % of all reads mapped to the reference protein and lncRNAs transcriptome (Table S5).

**Fig. 2:**
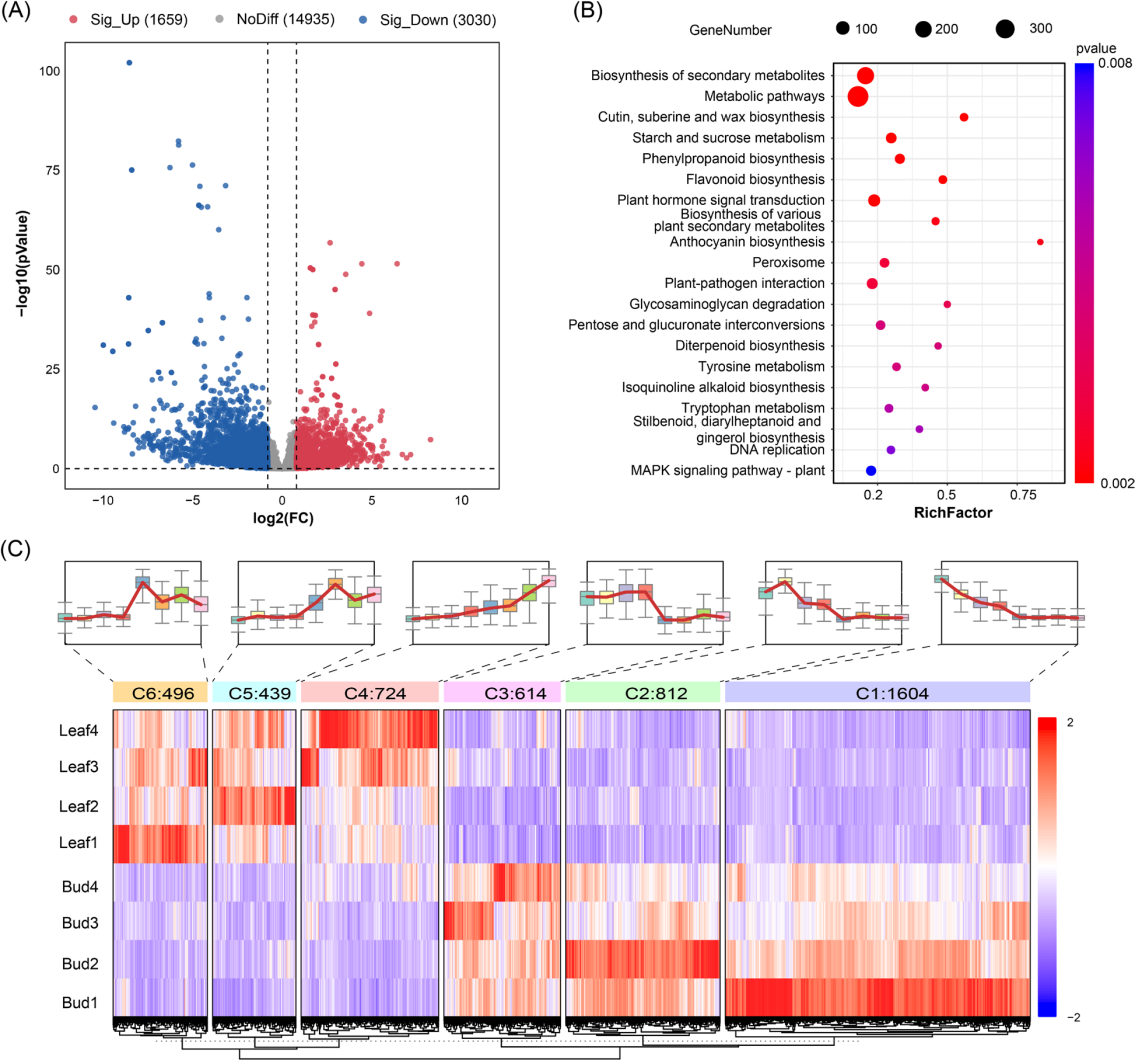
Differentially expressed genes (DEGs) analysis between Bud and Leaf groups. **A**Volcano plot of DEGs; (**B**) Scatter plot of the enriched KEGG pathways of DEGs; (**C**) Heatmap of DEGs expression patterns

Transcript samples from the same tissues exhibited strong and positive correlations, with Pearson correlation coefficients ranging from 0.89 to 0.98, whereas samples from different tissues displayed weaker correlations, with Pearson correlation coefficients ranging from 0.53 to 0.75 (Fig. S3A). PCA analysis also showed that the samples formed tissue-specific clusters, with the PC1 (34.4%) and PC2 (24.2%) being the most influential axes (Fig. S3B). In the transcriptome comparisons analysis, 4,689 DEGs were discovered between Bud and Leaf, comprising 1,659 upregulated and 3,030 downregulated genes in the Leaf (Fig. 2A, Table S6).

TFs are essential regulators of gene transcription in plant development. In this study, a total of 341 differentially regulated TFs were identified, representing 40 TFs families (Table S7). The results showed that the majority of these TFs were downregulated in Leaf. Among the TF families, ERF, bHLH, MYB, NAC and WRKY families exhibited the most abundant DEGs. In addition, the specific members of GATA, Dof and NAC were identified as significant DEGs with notably high fold-change values. Those TFs are likely to have played important roles in leaf development (Fig. S3C).

To gain insights into the biological functions and gene interactions of the DEGs, 2,116 out of 4,689 DEGs were successfully annotated in the GO database. The results showed that 21 GO terms were assigned to Biological Process, with the most highly abundant terms being cellular process (1,279) and single-organism (1,226; Fig. S4A). The terms of cell part (1683), cell (1,683) and organelle (1,205) were the most enriched in Cellular Component. In the Molecular Function category, terms such as the catalytic activity (919), binding (638) and nucleic acid binding (272) were the most enriched (Fig. S4A). Furthermore, among the top 20 significantly enriched GO terms (corrected *P* value < 0.05), six processes related to Biological Process were expressed differently in Bud and Leaf. In particular, 15 DEGs were significantly enriched in cutin biosynthetic process (GO:0010143) with the highest enrichment ratio (78.9%). In Molecular Function category, DEGs were significantly enriched in three terms, including microtubule motor activity (GO:0003777), nucleic acid binding transcription factor activity (GO:0001071) and transcription factor activity (GO:0003700). Additionally, eleven DEGs group were closely related to Cellular Component category (Fig. S4B and Table S8).

In the KEGG enrichment analysis, some DEGs between Bud and Leaf were significantly (corrected *P* value < 0.05) enriched in the phenylpropanoid metabolism, including phenylpropanoid biosynthesis, flavonoid biosynthesis and anthocyanin biosynthesis (Fig. 2B). The top 20 significantly enriched pathways (corrected *P* value < 0.05) also included the biosynthesis of secondary metabolites, such as starch and sucrose metabolism, glycosaminoglycan degradation and diterpenoid biosynthesis. Importantly, the enrichment of biosynthesis pathways of cutin, suberine and wax were consistent with that found in the GO enrichment results.

Among the 8,021 lncRNAs, 603 were determined as differentially expressed-lncRNAs (DE-lncRNAs). To explore the interactions between DE-lncRNAs and DEGs, PCC analysis was performed based on the expression level of lncRNAs and mRNA across eight samples. The lncRNAs-mRNA pairs with PCC absolute values exceeding 0.95 and *P* value < 0.05 were considered as potential interacting partners. In total, 1,764 DEGs were predicted to be the potential interacting partners of 453 DE-lncRNAs. To clarify the functions of lncRNAs in response to leaf development, KEGG pathway analysis was performed with the potential interacting DEGs. Several glycometabolism pathways, including starch and sucrose metabolism and pentose and glucuronate interconversions, were found to be significantly enriched (corrected *P* value < 0.05). In addition, pathways related to the biosynthesis of secondary metabolites were significantly enriched (corrected *P* value < 0.05), such as cutin, suberine and wax biosynthesis, isoquinoline alkaloid biosynthesis, phenylpropanoid biosynthesis, anthocyanin biosynthesis (Fig. S4C). These results provided valuable insights for further investigation into how lncRNAs regulate leaf development by participated in pathways.

To characterize the relationships among DEGs, we conducted expression profile clustering on eight samples using K-Means method. All DEGs were clustered into six clusters, including three upregulated patterns and three downregulated patterns (Fig. 2C). We conducted KEGG pathway enrichment analysis to identify the metabolic pathway related to the six clusters. The downregulated DEGs that related with the starch and sucrose metabolism and pentose and glucuronate interconversions were mainly found in cluster 1 and cluster3. Glycometabolism pathways, including galactose metabolism, fructose and mannose metabolism and pentose phosphate pathway, were primarily enriched in cluster 5 and cluster 6, which exhibited an upregulated pattern. Flavonoid biosynthesis and phenylpropanoid biosynthesis were significantly enriched in cluster 2, indicating that DEGs related to phenylpropanoid metabolism pathway displayed a coordinated trend. Cluster 4 was associated with plant-pathogen interaction suggesting a role in the stress response, possibly related to cultivation practices since no plant protection treatments were applied during sampling (Fig. S5). The reliability of the RNA-seq data was validated by the qRT-PCR analysis with 18 differentially expressed genes involved in key metabolism pathway (Fig. S6). The expression pattern of the 18 genes shown by qRT-PCR was highly consistent with that shown by RNA-seq. Pearson correlation analysis showed that *R*^*2*^ was 0.89. Thus, the transcriptome data were highly reliable.

### Metabolic profiles of different development stage

**Fig. 3:**
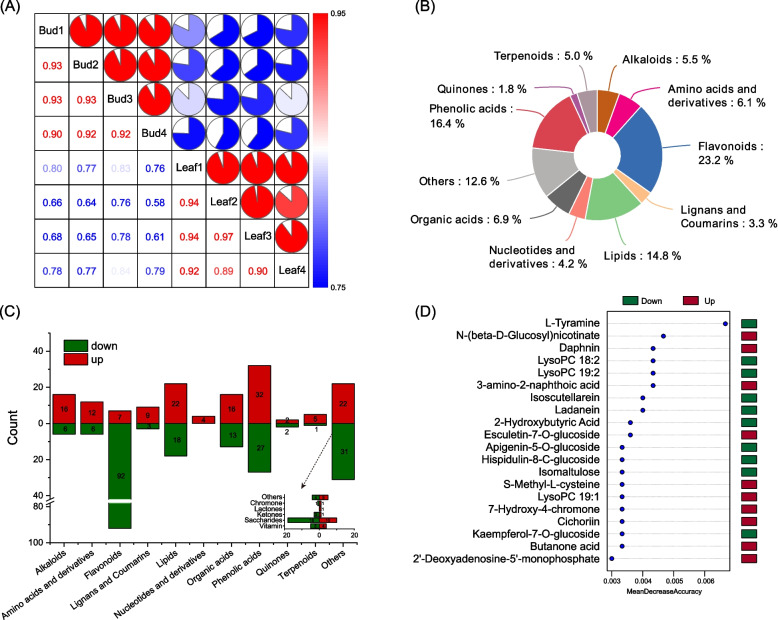
Profiling of metabolites in Bud and Leaf. **A**Correlation analysis between 8 samples; (**B**) Classifications and proportions of the metabolites identified from Bud and Leaf; (**C**) Classification, change and total number of differentially accumulated metabolites (DAMs) between Bud and Leaf; (**D**) Top 20 significant features identified by random forest

The metabolites produced by *P. juliae* Bud and Leaf were detected by using a UPLC-ESI-MS/MS system. The correlation analysis of the metabolomic data revealed that the four replicates of each group were clustered together, with Pearson correlation coefficients exceeding 0.89, indicating that the replicates had high consistency and reliability (Fig. 3A). PCA analysis further illustrated the overall metabolic differences among each group (Fig. S7A).

In total, 1,051 metabolites were successfully identified and annotated in all samples (Table S9). These metabolites could be classified into 11 different categories, with the majority falling under flavonoids (23.2%), phenolic acids (16.4%), and lipids (14.8%). In Bud, 1,006 metabolites were identified, while Leaf tissue had 1,008 identified metabolites (Fig. S7B). Bud and Leaf each had 43 and 45 unique metabolites, respectively. Further analysis revealed a total of 345 differentially accumulated metabolites between Bud and Leaf, with147 upregulated and 198 downregulated in Leaf. These metabolites were primarily comprised of flavonoids (99), phenolic acids (59), lipids (40), organic acids (29) and saccharides (28) (Fig. 3C, Fig. S7C and Table S10). To identify the most influential metabolites, random forest analysis revealed the top 20 metabolites which includes six flavonoids (Fig. 3D). Moreover, the PLS-DA highlighted 324 metabolite features with variable important in projection (VIP) values exceeding one (Fig. S7D).

The k-means analysis identified five primary clusters based on the standardized relative metabolite contents from eight samples (Fig. S8A). Cluster 4 (C4) and cluster 5 (C5) comprised 63 and 84 metabolites, respectively, with higher accumulation in Leaf. Cluster 1 (C1) was dominated by flavonoids, accounting for 76.8% of its composition, which exhibited a decrease in content. Flavonoids, phenolic acids, lipids, saccharides and organic acids were the five most abundant classes in cluster 2 and cluster 3 (Table S10).

**Fig. 4:**
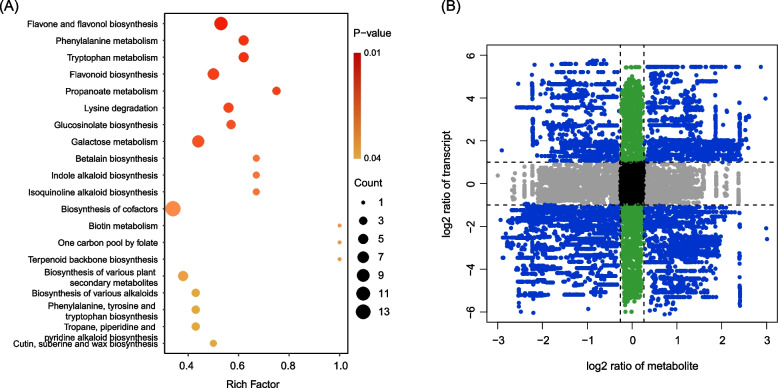
Analysis of DAMs between Bud and Leaf groups. **A**Scatter plot of the enriched KEGG pathways of DAMs; (**B**) The 9-quadrant plot of DAMs and DEGs with Pearson correlation coefficients > 0.9; the plot is divided from left to right and top to bottom into quadrants 1–9 with black dashed lines

In this study, we annotated and enriched the differential metabolites based on the KEGG database. KEGG pathway enrichment analysis revealed several significantly enriched metabolic pathways (corrected *P* value < 0.05), including flavone and flavonol biosynthesis, phenylalanine metabolism, tryptophan metabolism, flavonoid biosynthesis and propanoate metabolism (Fig. 4A). Notably, the pathways of flavonoid biosynthesis, isoquinoline alkaloid biosynthesis, tryptophan metabolism and cutin, suberine and wax biosynthesis were shared between metabolites KEGG enrichment analysis and DEGs enrichment analysis. This indicates a consistent and interconnected relationship between the transcriptomic and metabolomic pathways. In addition, the 9-quadrant plot of DAMs and DEGs highlighted the links between metabolites and genes. In quadrants 1, 3, 7 and 9, a total of 56,360 regulatory relationships were observed with PCC absolute value > 0.9 and *P* value < 0.05, suggesting a strong interconnection between DEGs and DAMs (Fig. 4B).

### Pathway analysis related to the nutrients and taste components

**Fig. 5:**
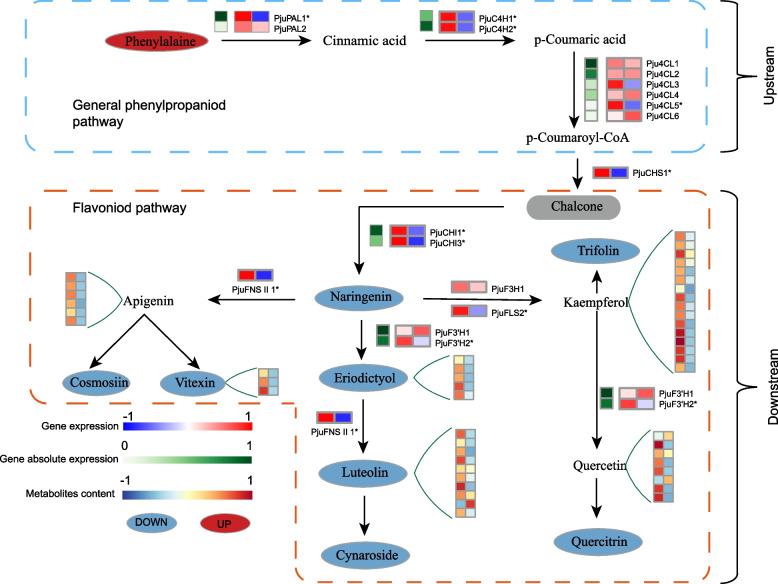
Flavonoid metabolic pathways during leaf development in *P. juliae*. The Red, blue and grey ellipse indicate significantly upregulation, significantly downregulation and not significant change metabolites, respectively. The derived DAMs changes of metabolites are shown in the green extension line with heatmap. Changes in annotated genes are presented as a heatmap at the corresponding locations

The KEGG enrichment analysis and the 9-quadrant plot both demonstrated the strong linkage between DEGs and DAMs, providing valuable insights into the molecular mechanisms. Flavonoids are the main nutrients and bioactive components in *P. juliae,* and exhibited markedly variation in content during leaf development. Based on transcriptome and metabolome data, we constructed a schematic flavonoids metabolism pathway (Fig. 5). Phenylalanine serves as the initial substrate for the phenylpropanoid metabolism pathway, which determined the metabolic flux of the entire pathway. Phenylalanine ammonia-lyase (PAL) is responsible for catalyzing the deamination reaction of phenylalanine to produce cinnamic acid, and is gateway and the rate-limiting enzyme of the phenylpropanoid metabolism pathway. In *P. juliae*, the content of phenylalanine in Leaf is significantly higher than in Bud, but the structural genes *PjuPAL1* significantly downregulated in Leaf. The expressions of *PjuC4H* encoding cinnamate 4-hydroxylase and *Pju4CL5* encoding 4-coumarate:coenzyme A ligase, which are involved in early enzymatic reactions, were significantly downregulated in Leaf. In addition, we found that the expression level of *PjuCHI* encoding chalcone isomerase was also significantly decreased, leading to a reduction in naringenin content. The downregulation of genes involved in these early enzymatic reactions appears to decrease the flux in the flavonoids biosynthesis pathways, thereby affecting flavonoid production.

In the context of flavonoids, the main accumulated compounds consist of glycosides and derivatives of apigenin, kaempferol, luteolin and Quercetin (Fig. S8B). In the biosynthesis pathways of flavonoids, some key branch point genes were characterized. The significant decrease in the expression of the *PjuFLS2* encoding flavonol synthase likely contributes to the reduction of glycosides and derivatives of kaempferol. Moreover, we observed that the *PjuF3’H2* gene encoding flavonoid 3’-hydroxylase was significantly downregulated in Leaf, which might be a critical factor directly influencing the accumulation of glycosides and derivatives of quercetin and eriodictyol. As for glycosides present and derivative of apigenin and luteolin, the significant downregulation of the *PjuFNSII1* encoding flavone synthase II appears to be responsible for the decrease observed in Leaf.

TFs play a crucial role in the regulating flavonoids biosynthesis in plants. To identify the TFs related to flavonoids biosynthesis in *P. juliae*, a co-expression network was constructed based on the correlations between flavonoids related structural DEGs and TFs. This analysis revealed 25 different TF families related to flavonoids biosynthesis in *P. juliae* (Fig. S8C and Table S11). The top five families identified were MYB, HD-ZIP, bHLH, ERF and TCP. These TFs are likely involved in the regulation of flavonoids metabolism and plant growth and development. In addition, we explored the potential regulatory role of the lncRNAs in this network to understand the interactions between lncRNAs, genes, and TFs. We found that 178 lncRNAs were significantly correlated (*P* value < 0.05) with DEGs and TFs involved flavonoids biosynthesis. In the lncRNAs-genes-TFs network, it was notable that three MYB TFs (*PjuMYB44*, *PjuMYB46* and *PjuMYB56*) had the most abundant connections, acting as key nodes connecting DEGs and lncRNAs (Fig. S8C). The co-expression networks illustrated the central role of MYB TFs regulating flavonoids biosynthesis.

**Fig. 6:**
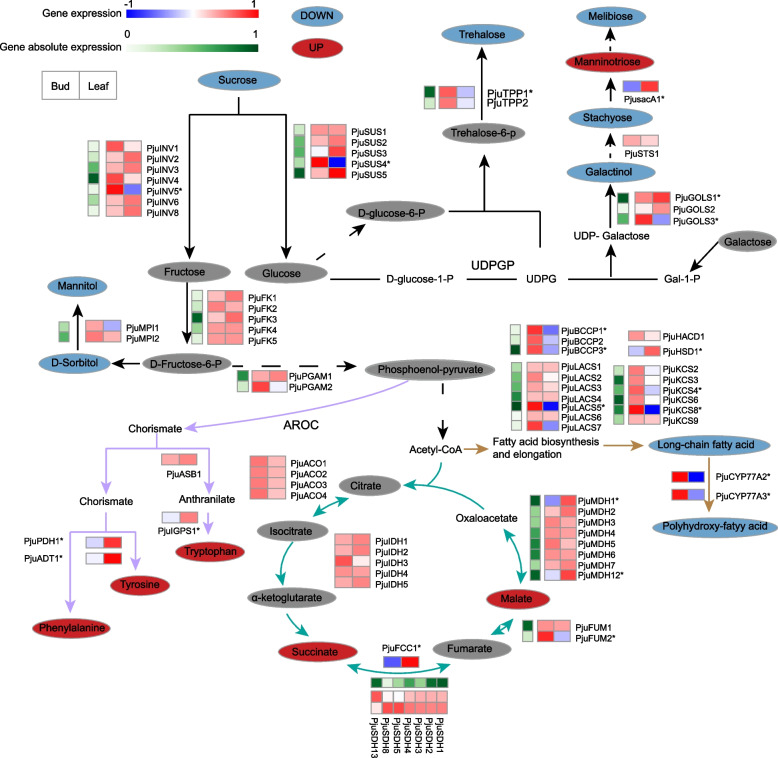
The carbohydrate biosynthesis pathway and the downstream pathways during leaf development in *P. juliae*. The Red, blue and grey ellipse indicates significantly upregulation, significantly downregulation and not significantly change metabolites, respectively. The derived DAMs changes of metabolites also shown in the green extension line with heatmap

The metabolic pathways related to carbohydrates and downstream processes that produce sugars, organic acids and amino acids often determine flavor, nutritional composition, and leaf development in plants. A key metabolic network was constructed based on transcriptome and metabolome data, encompassing sugar metabolism, cutin metabolism, tricarboxylic acid cycle (TCA cycle), and aromatic amino acids metabolism (Fig. 6). In the sugar pathway, the content of compounds like trehalose, stachyose, melibiose, mannitol, D-sorbitol and galactinol were significantly lower in the Leaf than in the Bud, while the manninotriose was significantly higher. The expression levels of the *PjuTPP1* encoding trehalose-6-phosphate phosphatase and *PjusacA1* encoding β-fructofuranosidase were consistent with the observed compound content. Malate and succinate were the main components of organic acids in *P. juliae*. These compounds significantly accumulated more in Leaf. This increase was associated with the significant upregulation of DEGs like *PjuFCC1* encoding fumarate reductase (which accelerated the reduction of fumarate to succinate) and *PjuMDH1* and *PjuMDH12* encoding malate dehydrogenase (involved in malate biosynthesis). In the aromatic amino acids metabolism, the significant accumulation of phenylpropane, L-phenylalanine, tyrosine and tryptophan in Leaf was linked to the significant upregulation of DEGs such as *PjuPDH1* encoding prephenate dehydrogenase and *PjuADT1 *encoding arogenate dehydratase, which are related to biosynthesis. In addition, several DEGs involved in long-chain fatty acid and polyhydroxy fatty acid metabolism, such as *BCCP* genes (encoding biotin carboxyl carrier protein, *PjuBCCP1* and *PjuBCCP3*), *ACS* gene (encoding acyl-CoA synthetase, *PjuLACS5*), *KCS* genes (encoding ketoacyl-CoA synthase, *PjuKCS4* and *PjuKCS8*) and *CYP* genes (encoding cytochrome P450 enzymes, *PjuCYP77A2* and *PjuCYP77A3*), were significantly downregulated in Leaf. They may contribute to the corresponding adjustment of cutin in different development stages.

## Discussion

The complexity of genome assembly and the limitation of gene information are significant impediments to investigate the molecular mechanisms governing critical biological processes related to quality development in many plants with economic value. In the present study, we provided the first comprehensive, high-quality, full-length transcriptome of the *P. juliae*. These data hold great potential for advancing molecular-level research on *P. juliae*. Based on the high-quality transcripts, we integrated metabolite profiles with NGS transcriptome data to explore the constituents contributing to nutrients and taste, as well as the molecular regulations underlying these processes.

Studying nutrient quality not only helps us to select an optimal breeding program by exploring the most promising vegetable genotypes, but also contributes to improve the vegetables quality, health-promoting components, commodity status, and market value [37]. Recent researches have shown that the active ingredients within *Primulina* extract possess anti-inflammatory properties, suppress tumor cells growth, regulate blood sugar levels, and render other physiological functions [15, 38–40]. In this study, a total of 1,051 metabolites were identified in the *P. juliae*, spanning various categories such as flavonoids, phenolic acids, lipids, organic acids, amino acids and derivatives, alkaloids, terpenoids, nucleotides and derivatives, lignans and coumarins, quinones and others metabolites (Fig. 3B). Flavonoids, phenolic acids, alkaloids and terpenoids are known for their anti-inflammatory, antiviral, antibacterial and other effects [41–44]. In this study, flavonoids content varied during developmental stages, piquing our research interest. Among DAMs in the flavonoid category, we have identified 31 active flavonoids using the TCMSP database (Table S12). Except for lonicerin and ampelopsin, the levels of other flavonoids exhibited a substantial decrease during the leaf development. The screening of these active flavonoids can provide a valuable reference for the development of functional foods. In the flavonoid metabolic pathway, the PAL enzyme is considered to be a pivotal regulatory component, acting as a rate-limiting enzyme in polyphenol metabolism and serving as the initial enzyme in the first phase of flavonoid synthesis [45]. In *P. juliae*, phenylalanine content was significantly increased in Leaf, but the significant downregulation of *PAL* genes may have limited the metabolic flow, resulting in the downregulation of most downstream products. Meanwhile, other structural genes related to biosynthesis also displayed significant downregulation. *PjuMYB44*, *PjuMYB46* and *PjuMYB56* were identified as hub genes with strong connectivity in the turquoise module (Fig. S8C), indicating their vital roles in the flavonoids pathway. Overall, these results indicated that these MYBs are important TFs affecting the expression of genes responsible for synthesizing flavonoid components. Therefore, the specific mechanisms by which these candidate TFs regulate the related structural genes in *P. juliae* deserve further investigation. In addition, some sugars, phenolic acids, alkaloids, and terpenoids are also important nutrients. We noticed that phenolic acids also exhibited significant developmental differences. Trehalose and mannose have important effects in resisting cadmium poisoning, anti-apoptosis, and protecting neurons [46–48]. In our study, the expression levels of *PjuTPP1* and *PjusacA1* genes were consistent with the trehalose and mannose content, suggesting that these two genes play key roles in the pathway (Fig. 6). Further research should be conducted on functional vegetables to assess the antibacterial and food nutrition enhancement properties for enriching the edible value of *P. juliae*.

Taste characteristics are key traits in crop domestication, which determine consumer acceptance of flavor. The sweetness of the vegetable is mainly determined by its soluble sugar content, and the acidity is controlled by organic acids. The sugar/acid ratio determines the overall taste of vegetables. During the leaf development *P. juliae*, there was an increase in the content of organic acids, specifically malate and succinate. The content of sucrose in Leaf was significantly lower than in Bud. Except for manninotriose, the content of sugar compound in the metabolic pathway either significantly decreased or did not show significant changes.

Hexoses-P, derived from glucose and fructose, can be used for the synthesis of organic acids through glycolysis and the TCA cycle. The NAD-MDH enzyme controls the reversible reaction between malate and oxaloacetate, and it serves various functions in plant cells as well [49]. In the TCA cycle, the synthesis of malate is regulated by bidirectional metabolism. Fumarate is transformed into malate by the enzyme fumarase [50]. Malate dehydrogenase, a widely distributed enzyme in animals and plants, catalyzes the interconversion of malate and oxaloacetate, influencing multiple metabolic pathways. In alfalfa (*Medicago sativa*) [51], wheat (*Triticum aestivum*) [52] and cordifolia (*Aptenia cordifolia*) [53], MDH primarily catalyzes the conversion from oxaloacetate to malate. In our study, two members of the *MDH* gene family, *PjuMDH1* and *PjuMDH12*, are likely pivotal in regulating malate content. In the case of succinate, genes encoding fumarate reductase, an enzyme converts fumarate into succinate, significantly upregulated during the leaf development in *P. juliae*. Although the *PjuSDH* gene encoding succinate dehydrogenase did not change significantly, the upregulation of *PjuFCC1* may play a key role in succinate accumulation in *P. juliae* leaf. These variations in sugar and organic acid content significantly impact the vegetable taste. In the future, it is necessary to evaluate the specific tastes with electronic tongue technology.

The plant cuticle is an extracellular hydrophobic layer that covers the aerial epidermis of all land plants. Cuticle played an essential role in limiting transpiration [54]. Cutin is a major component of the leaf cuticle. *Primulina juliae* is known for having leaves with high water content, which contributes to its crispy texture, making it suitable for use in cold dishes. Our study revealed that long-chain fatty acids and polyhydroxy-fatty acids were decreased their content during the development of *P. juliae*. The reduction of these components affected properly formed and structured cutin matrix, which is critical for the cuticle’s function as a barrier to limit water loss [55]. Furthermore, genes related to cutin biosynthesis were also found to be significantly downregulated. These genes included *PjuBCCP1*, *PjuBCCP3*, *PjuLACS5*, *PjuKCS4*, *PjuKCS8*, *PjuCYP77A2*, and *PjuCYP77A3*.

Tryptophan and phenylalanine, derived from the chorismate pathway, are essential amino acids known for their strong bitterness in previous studies [56, 57]. In *P. juliae*, we observed a significant accumulation of tryptophan and phenylalanine during leaf maturation. Our data suggests that *PjuIGPS1* encoding indole-3-glycerol phosphate synthase and *PjuADT1* are important candidate genes involved in the biosynthesis of tryptophan and phenylalanine.

## Conclusions

**Fig. 7:**
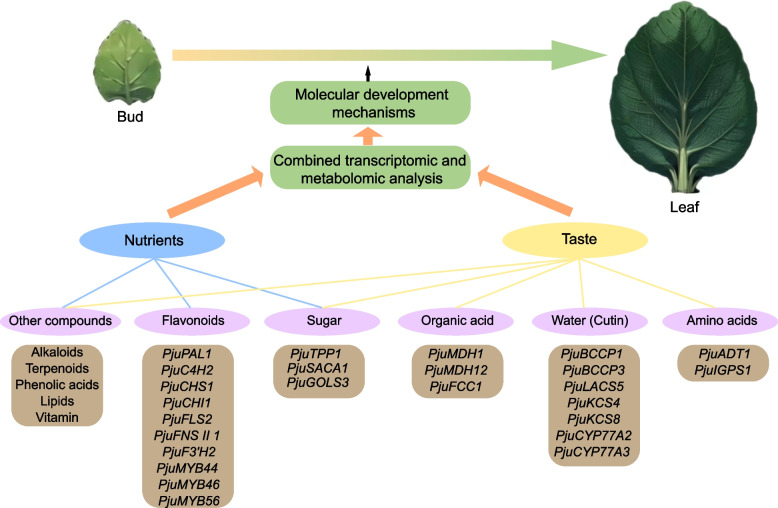
Model of the molecular mechanism of the nutrients and taste components development

The present study provided the first set of full-length transcriptome in *P. juliae*. Through a comprehensive transcriptomic and metabolomic analysis of young and mature leaves, we have revealed molecular mechanisms in nutrients and taste components development between different stages (Fig. 7). Our results indicated that some organic acids (malate and succinate) and amino acids (tryptophan and phenylalanine) related with taste increased significantly as the leaves matured. However, most of the flavonoids, which are the main nutrients in *P. juliae*, were significantly downregulated. This means that appropriate harvesting times and sorting strategies should be applied in production. In addition, key candidate genes related to metabolites biosynthesis pathway were screened out by the comprehensive analysis. This study offers a solid theoretical foundation and valuable practical guidance for enhancing molecular breeding and quality improvement in *P. juliae*.

### Supplementary Information

Additional file 1: Figure S1-S8.

Additional file 2: Table S1-S12.

## Data Availability

The datasets generated and analyzed during the current study are available in National Center for Biotechnology Information (NCBI) BioProject database under accession number PRJNA1032441.

## Abbreviations

PAL: Phenylalanine ammonia-lyase
C4H: Cinnamate 4-hydroxylase
4CL: 4-coumarate:coenzyme A ligase
CHS: Chalcone synthase
CHI: Chalcone isomerase
F3’H: Flavonoid 3’-hydroxylase
F3H: Flavanone 3-hydroxylase
FNS: Flavone synthase
FLS: Flavonol synthase
INV: Invertase
SUS: Sucrose synthase
FK: Fructokinase
AROC: Chorismate synthase
ASB1: Anthranilate synthase beta subunit
PDH: Prephenate dehydrogenase
IGPS: Indole-3-glycerol phosphate synthase
ADT: Arogenate dehydratase/prephenate dehydratase
TPP: Trehalose-6-phosphate phosphatase
STS: Stachyose synthase gene
GOLS: Galactinol synthase
PGAM: Phosphoglycerate mutase
ACO: Aconitase
SDH: Succinate dehydrogenase
FCC: Fumarate reductase
FUM: Fumarate hydratase
MDH: Malate dehydrogenase
BCCP: Biotin carboxyl carrier protein
ACS: Acyl-CoA synthetase
HACD: 3-hydroxyacyl-CoA dehydratases
HSD: Hydroxysteroid dehydrogenase
KCS: Ketoacyl-CoA synthase
MPI: Mannose-6-phosphate isomerase
D-Fructose-6-P: D-Fructose-6-phosphate
D-glucose-6-P: D-Glucose 6-phosphate
D-glucose-1-P: D-Glucose-1-phosphate
UDPG: Uridine diphosphate glucose
Gal-1-P: Galactose-1-phosphate
Trehalose-6-p: Trehalose-6-phosphate
