# Supplementary material for: Combined full-length transcriptomic and metabolomic analysis reveals the molecular mechanisms underlying nutrients and taste components development in Primulina juliae
Source: BMC Genom Data. 2024 May 23;25:46. doi: 10.1186/s12863-024-01231-z (PMC11112898; doi:10.1186/s12863-024-01231-z)

**Supplementary Figures**

**Title:** Combined full-length transcriptomic and metabolomic analysis reveals the molecular mechanisms underlying nutrients and taste components development in *Primulina juliae*

**Authors:** Yi Zhang, Endian Yang, Qin Liu, Jie Zhang, Chen Feng*

**Additional file 1:**

Fig. S1 Leaf phenotypes changes during leaf development. L1, L2, L3 and L4 represents the first leaf, the second leaf (Bud), the third leaf and the fourth leaf (Leaf), respectively. Scale bar = 1 cm.


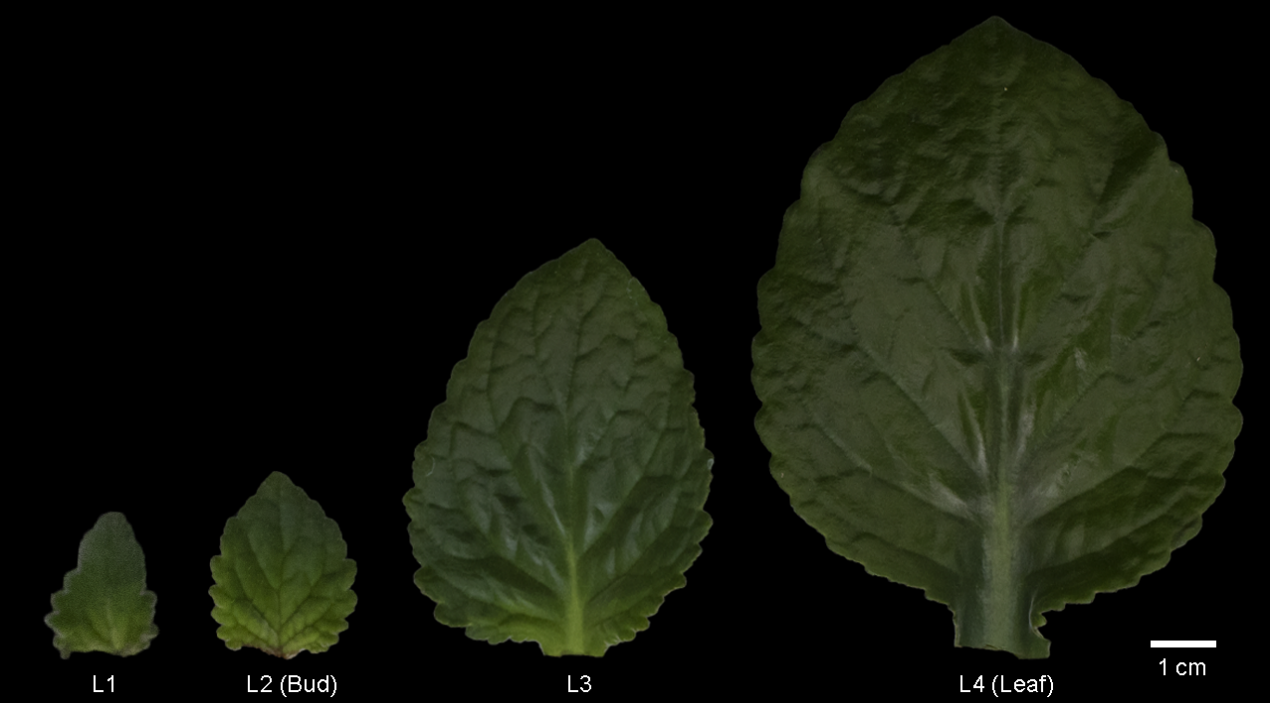


Fig. S2 Length distribution of the subreads. (A) Length distribution of raw subreads; (B) Length distribution of circular consensus sequencing (CCS) subreads; (C) Length distribution of full-length non-chimeric (FLNC) subreads; (D) Length distribution of cluster subreads.


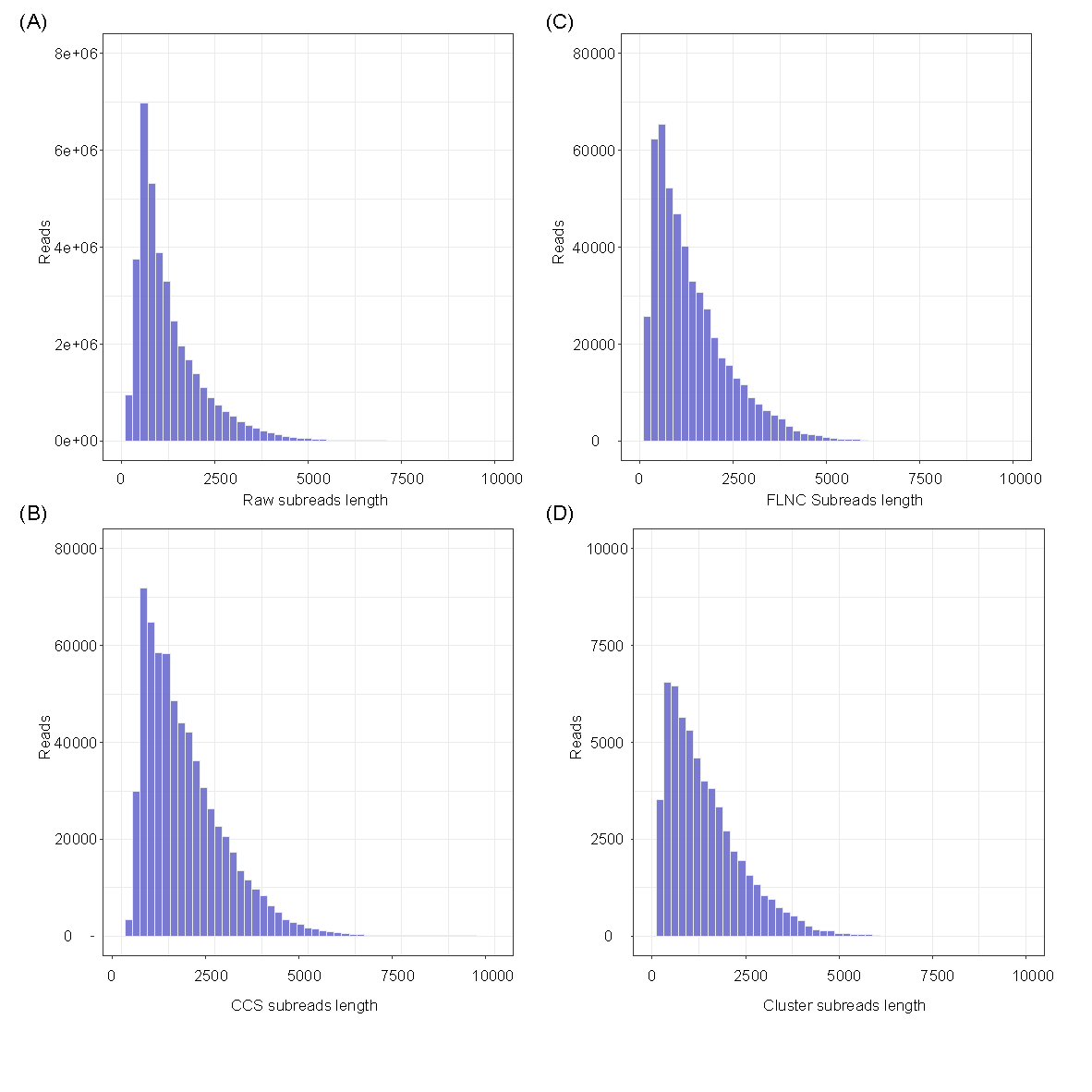


Fig. S3 Multivariate statistical analysis of transcriptomic. (A) Pearson correlation analysis between 8 transcriptomic samples; (B) Principal component analysis among 8 transcriptomic samples; (C) Scatter plot of TFs classification from DEGs.


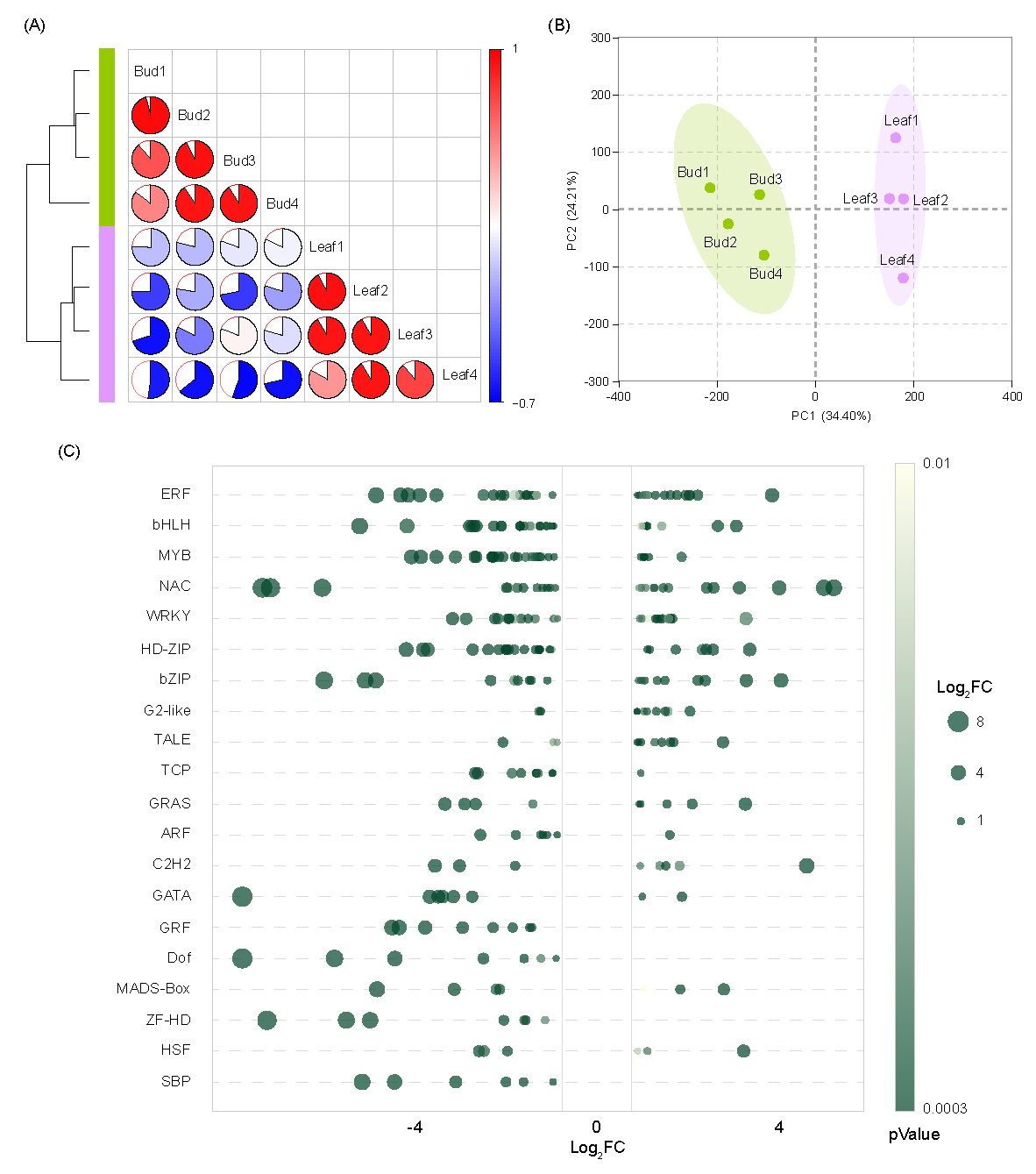


Fig. S4 Enrichment analysiss of GO and KEGG. (A) Distribution of GO terms for all annotated DEGs; (B) GO enrichment results of the DEGs; (C) KEGG enrichment analysis of the DEGs related to differentially expressed-LncRNAs (DE-LncRNAs).


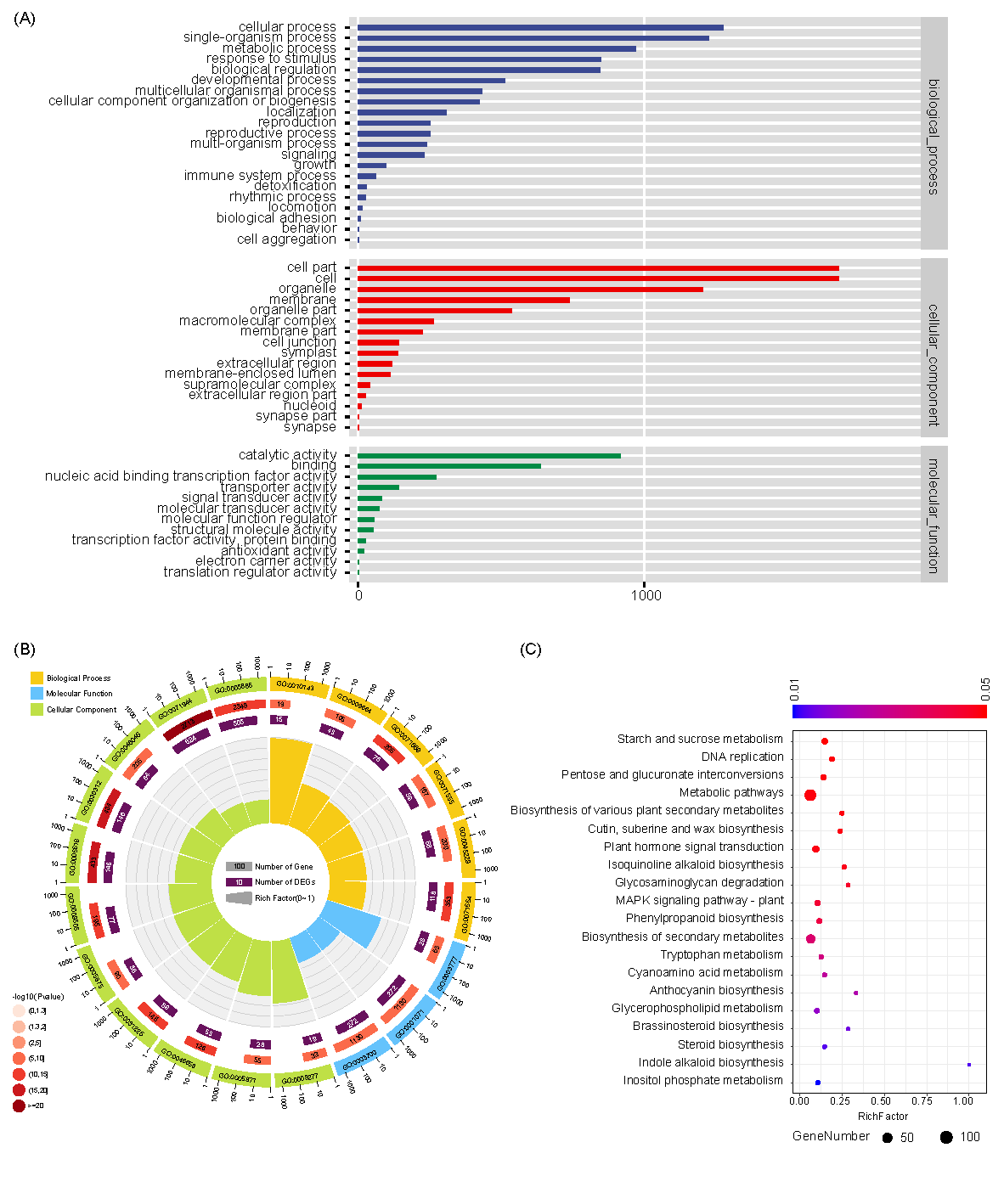


Fig. S5 KEGG enrichment analysis of each cluster based on DEGs expression. C1, C2, C3, C4, C5 and C6 indicates cluster1, cluster2, cluster3, cluster4, cluster5 and cluster6.


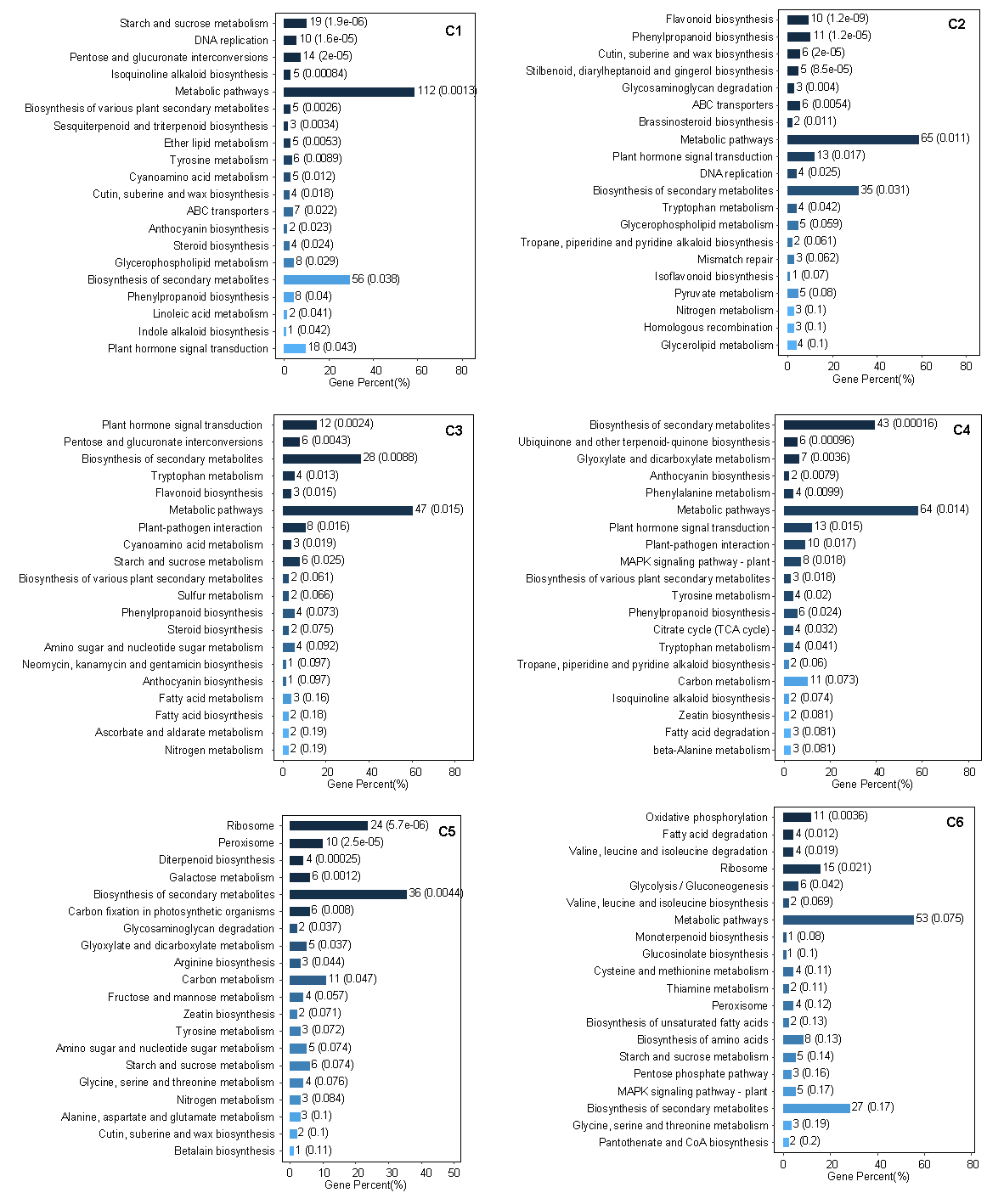


Fig. S6 qRT-PCR validations of expression patterns of DEGs related to metabolism pathway. Values shown are mean ± SD of 3 replicates.
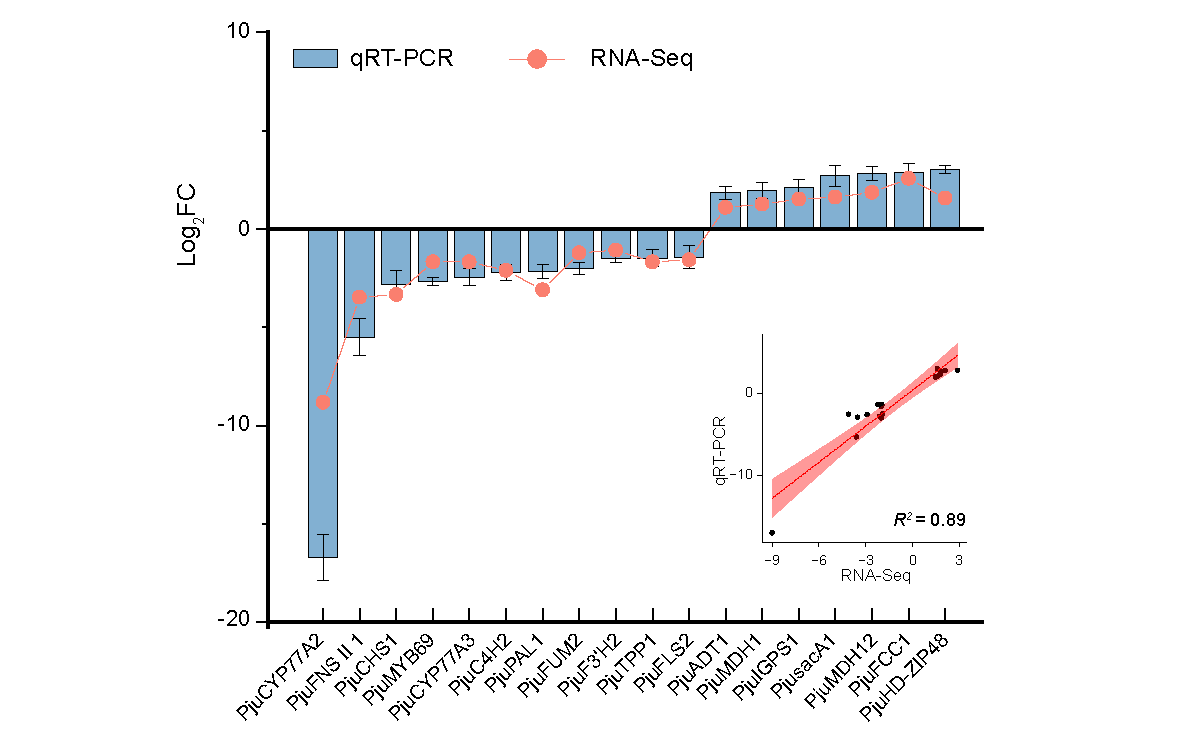


Fig. S7 Multivariate statistical analysis of metabolic. (A) Principal component analysis among 8 transcriptomic samples; (B) Venn diagram of the detected metabolites in Bud and Leaf; (C) Volcano plot of DAMs; (D) VIP scores of top 20 DAMs; (E) Scatter plot of DAMs change based on Log2(FC) value.


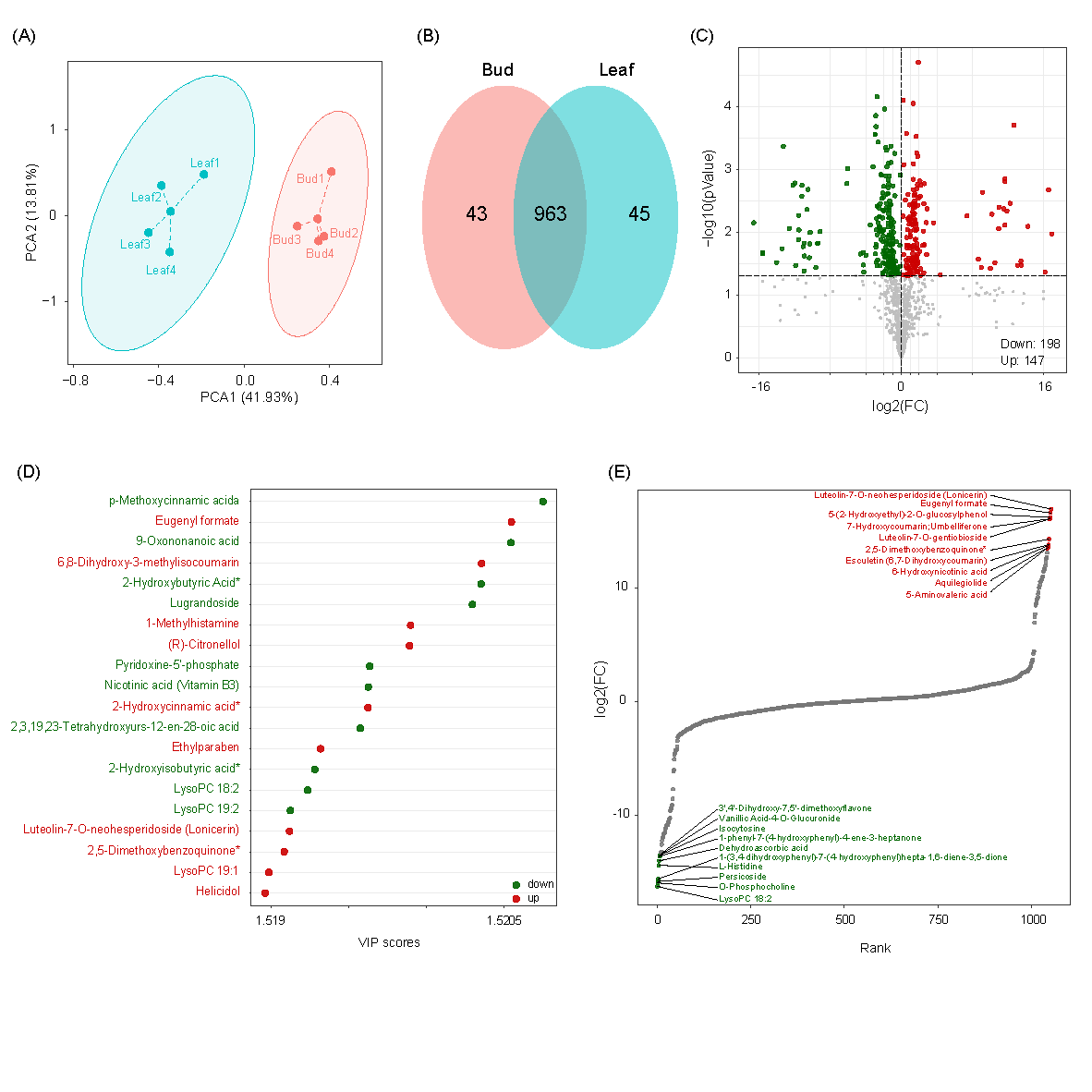


Fig. S8 Analysis of relative content of metabolites and co-expression network analysis. (A) Heatmap of DAMs relative content change patterns; (B) Heatmap of relative content from differentially accumulated flavonoids; (C) Co-expression networks between the lncRNAs-genes-TFs network involved in flavonoids biosynthesis pathway.


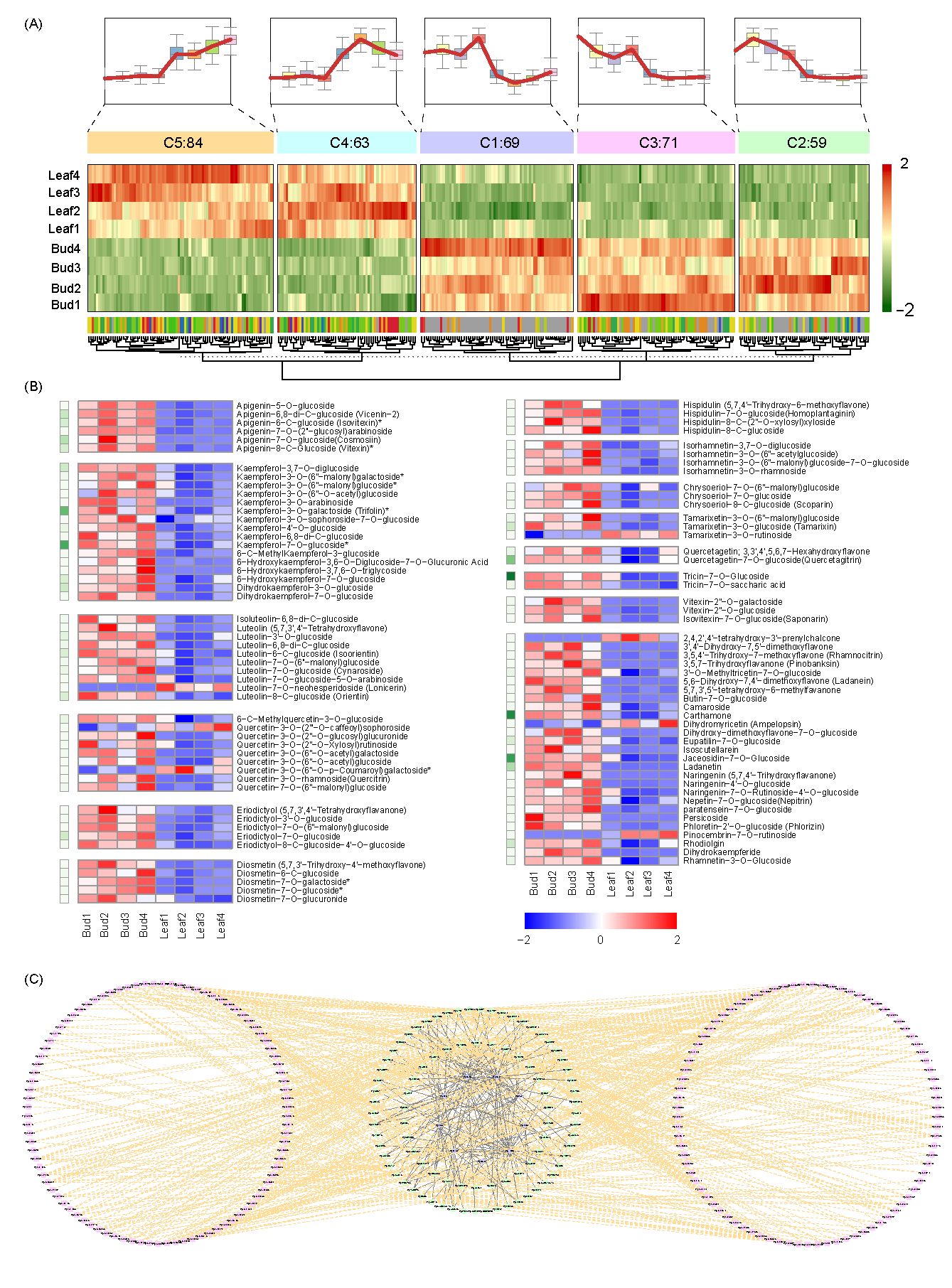

Supplement: Supplementary file 1 — Additional file 1: Figure S1-S8. [file 12863_2024_1231_MOESM1_ESM.docx]
